# Treatment Modalities for Refractory-Recurrent Tenosynovial Giant Cell Tumor (TGCT): An Update

**DOI:** 10.3390/medicina60101675

**Published:** 2024-10-12

**Authors:** Vasiliki Dania, Nikolaos A. Stavropoulos, Panayiotis Gavriil, Ioannis Trikoupis, Panagiotis Koulouvaris, Olga D. Savvidou, Andreas F. Mavrogenis, Panayiotis J. Papagelopoulos

**Affiliations:** First Department of Orthopedic Surgery, School of Medicine, National and Kapodistrian University of Athens, “ATTIKON” University General Hospital, 12462 Athens, Greece

**Keywords:** giant cell tumor, tenosynovial giant cell tumor, localized type, diffuse type

## Abstract

*Background and Objectives*: Tenosynovial giant cell tumor (TGCT) is a rare, locally aggressive, benign neoplasm arising from the synovium of joints, tendon sheaths, and bursa. There are two main subtypes of TGCT: localized-type TGCT(L-TGCT) and diffuse-type TGCT (D-TGCT). While surgical excision is still considered the gold standard of treatment, the high recurrence rate, especially for D-TGCT, may suggest the need for other treatment modalities. *Materials and Methods*: This study reviews current literature on the current treatment modalities for refractory-relapsed TGCT disease. *Results*: The gold standard of treatment modality in TGCT remains surgical excision of the tumor nevertheless, the elevated recurrence rate and refractory disease, particularly in D-TGCT indicates and underscores the necessity for additional treatment alternatives. *Conclusions*: TGCT is a benign tumor with inflammatory features and a potential destructive and aggressive course that can lead to significant morbidity and functional impairment with a high impact on quality of life. Surgical resection remains the gold standard current treatment and the optimal surgical approach depends on the location and extent of the tumor. Systemic therapies have been recently used for relapsed mainly cases.

## 1. Introduction

Tenosynovial giant cell tumor (TGCT) is an infrequent non-malignant inflammatory mesenchymal tumor that emerges from the synovium of the joint, tendon sheath, and bursa. It can manifest as either a single nodule (localized) or as multiple noduli (diffuse) [1,2].

The first documented case of TGCT was reported by French surgeon M. Chassaignac in 1852. Subsequently, in 1941, Jaffe et al. documented twenty cases with soft tissue lesions identified as pigmented villonodular synovitis (PVNS) [2,3]. Granowitz et al. (1976) proposed two distinct forms of TGCT: localized and diffuse types [4].

According to the 2013 World Health Organization classification, the term localized TGCT refers to a giant cell tumor of the tendon sheath (GCTTS) and nodular tenosynovitis, whereas diffuse TGCT refers to a diffuse-type giant cell tumor and PVNS [1].

At a 2022 consensus meeting in Germany, TGCT was further classified into two distinct forms: nodular TGCT (N-TGCT) and diffuse TGCT (D-TGCT). Nodular TGCT corresponds to localized TGCT [5].

The disease has a characteristic monoarticular progression. N-TGCT usually involves the small joints of the hand and toes, with a predilection for the flexor tendons of the fingers and the distal phalanx and, less frequently, the synovial lining of a bursa or joint. Usually, N-TGCT manifests as a solidary palpable lesion emerging close to tendons or interphalangeal joints and occasionally can erode bone or involve the skin [6]. Among soft tissue tumors of the hand, N-GCCT is the second most prevalent, following ganglion cysts [7]. N-TGCT can affect large joints like the knee but this does not occur frequently. There is a low incidence of N-TGCT of the elbow [8].

Further to the aforementioned, D-TGCT, often involving larger joints, can either be located intra-articularly, with characteristic joint effusion and involvement of articular surfaces, or extra-articularly, possibly extending to the soft tissue envelope as well. Clinical symptomatology may include but is not limited to cystic formations, cartilaginous lesions of variant depth and size, and possible limitation of range of motion [5].

## 2. Epidemiology

The incidence rates for N-TGCT in studies conducted in Denmark and the Netherlands ranged from 30.3 to 34 per million person-years for peripheral digits and 11 per million person-years for extremities including the knee, hip, and ankle. The incidence rates of D-TGCT ranged from 5 to 8.4 per million person-years [9,10]. Furthermore, Ehrenstein et al. revealed a prevalence of 44.3 per 100,000 individuals for N-TGCT and 11.5 per 100,000 for D-TGCT [10]. The documented occurrence rate for M-TGCT is less than 1 in 1000 instances per year, and the likelihood of metastasis is around 50% [10,11,12,13,14,15,16].

TGCT in pediatric patients is rare. In the Netherlands, the reported incidence rates for N-TGCT (excluding digits) and D-TGCT were 2.86/1,000,000 and 1.30/1,000,000, respectively. The knee is the most commonly involved joint, particularly affecting females. Compared to adults, the 2.5-year recurrence-free survival rate (RFS) after surgical treatment in children is 85% vs. 89% in N-TGCT and 53% vs. 56% in D-TGCT [17].

Both subtypes can manifest at any age but have a higher prevalence among a young population aged 30–50 years, with a slightly higher occurrence in female patients compared to male patients [1].

The recurrence rate is lower in N-TGCT (9–14%) than in the diffuse type, for which it is 23–72% and remains significantly high. For D-TGCT, the 1- and 5-year recurrence-free survival rates are 69% and 32%, respectively [14,15].

## 3. Clinical Presentation

The clinical presentation differs significantly based on the anatomical location and the disease progression. TGCT is a monoarticular disease and because of the non-specific functional symptoms it can imitate other monoarticular pathologies.

Clinically, the predominant symptom in N-TGCT is a painless enlarged joint with insidious onset. Despite its gradual growth, symptoms include pain, discomfort, and a mechanical type of block in the affected joint. In superficial locations, clinical examination reveals a soft palpable mass [8,18].

**Figure 1:**
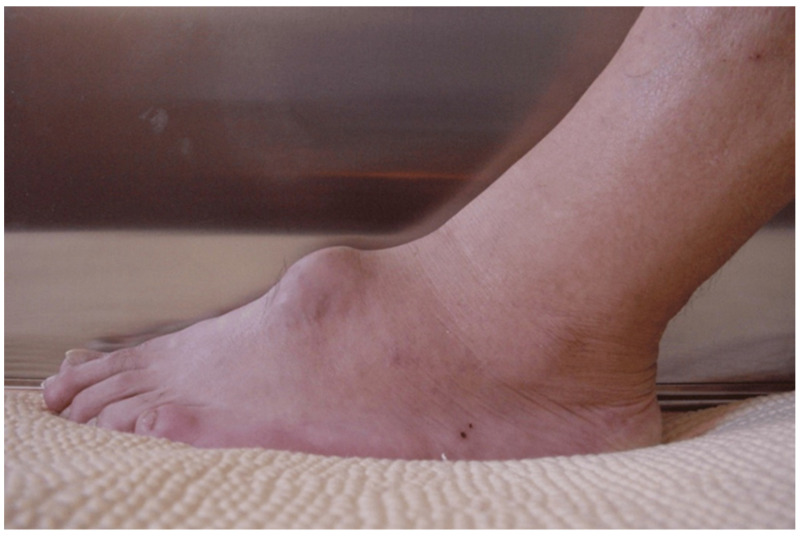
Painless swelling on a foot dorsal aspect. Lateral view on clinical examination.

Compared to N-TGCT, D-TGCT, which usually involves large joints, with the knee the most predominantly affected joint, has more concerning presenting symptoms, like pain, swelling, and stiffness. Physical examination reveals signs of irritation, including redness, swelling in the joints, tenderness, and eventually decreased ability for movement in the affected area. As the disease progresses, recurring hemarthrosis may aggravate joint stiffness and result in significant joint damage, greatly affecting daily activities [8,18,19] (Figure 1).

## 4. Imaging

While magnetic resonance imaging (MRI) is the most precise imaging technique to detect and evaluate TGCT, plain X-ray is the first step. Plain radiographs reveal a benign-appearing circumscribed soft tissue shadow in 50% of cases of giant cell tumor of the tendon sheath. Radiographs can also demonstrate cortical erosion of the bone due to the proximity of the adjacent mass on the cortex. True bone invasion is not typical and suggests an aggressive neoplasm. So, X-rays should be obtained to rule out any underlying bony involvement and to rule out calcifications, which are rarely seen in TGCT. Bone density and joint space are preserved until the late stages [5,20,21].

The MRI protocol should include T1-weighted, T2-weighted, and fluid-sensitive sequences with gadolinium enhancement. MRI findings, which are nearly pathognomonic for TGCT, include low or intermediate signal intensity on T1-weighted sequences, low signal intensity on T2-weighted images, and a blooming artifact due to iron in the hemosiderin deposition, which occurs prominently in intra-articular D-TGCT and is highly diagnostic of TGCT disease [22]. On the other hand, D-TGCT demonstrates multilobulated lesions with irregular margins and synovial thickening and villous projections. D-TGCT also presents frequently with extensive joint involvement, bone erosions, joint effusion, and blooming artifacts. Extra-articular D-TGCT is characterized by infiltrative growth pattern with lesions in the peri-articular soft tissues, muscle, and subcutaneous tissue. Osseous erosions and cysts usually are observed in joints such as the hip [5,23,24]. Malignant TGCT has similar MRI features to benign TGCT [5].

**Figure 2:**
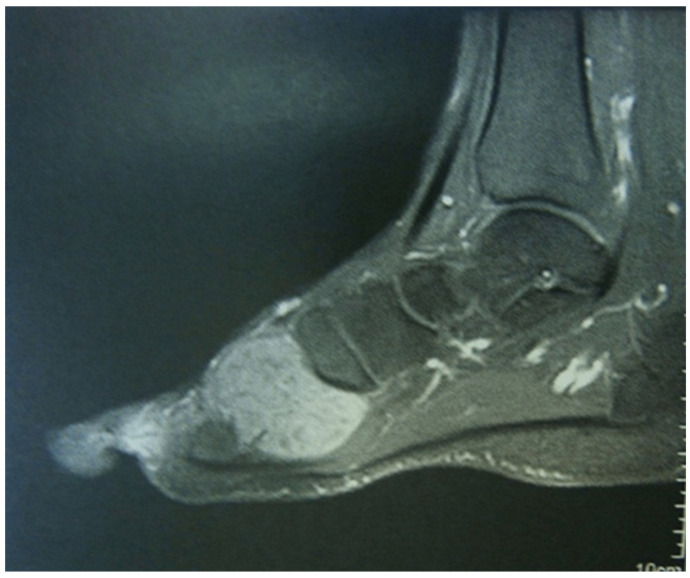
T1 weighted sequence with fat signal suppression post-gadolinium.

In N-TGCT, MRI findings include a distinct, focal lesion with a blooming artifact having lower sensitivity. Joint effusion is typically absent and intralesional areas of high T2 signal are related to necrosis [22,25] (Figure 2).

## 5. Histopathology

TGCT has typical morphology features. N-TGCT presents as a lobulated tumor with a variably yellow, whitish, or tan cut surface, and the diffuse type in the intra-articular form has a villous presentation and in the extra-articular form indicates a multinodular profile with a variegated cut surface [5].

TGCT, in general, is a fibro histiocytic tumor with two cell types for the mononuclear cells: small histiocytic-like cells and larger epithelioid cells [6].

The histopathologic features consist of mononuclear cells, multinucleated osteoclast-like giant cells, foamy (lipid-loaded) macrophages, inflammatory cells, siderophages or cells (containing a rim of haemosiderin granules), fibroblast-like synoviocytes (FLS) and stromal hyalinization. Osteoclast-like giant cells may be sparse or absent. The difference between N-TGCT and D-TGCT is that D-TGCT presents mostly as an infiltrative mass with fewer multinucleated giant cells, lower stromal hyalinization, and more xanthoma cells [22,26,27,28,29,30,31].

In the synovial fluid of affected joints, various inflammatory factors such as IL-1 and TNF-a are usually seen [32]. In 1941, Jaffe et al. suggested that TGCT has an inflammatory origin but in 2006 West et al. discovered that TGCT is characterized by chromosomal aberrations, suggesting a neoplastic origin [2,33]. These chromosomal translocations involve 1p11-13 and a subset of these fusions occurs with 2q37, where the colony-stimulating factor (CSF-1), also known M-CSF1(macrophage-CSF1) gene, and the collagen-type VI alpha-3 (COL6A3) gene, respectively, are located. This rearrangement, t(1;2) (p13;q37), which results in the formation of a COL6A3-CSF1 fusion product, leads to overexpression of CSF-1 [33,34]. This fusion pattern is present only in a subset of patients where other patterns of fusion have been described and CSF1 is typically upregulated regardless of fusion status [33,35].

CSF-1 promotes macrophage differentiation, proliferation, survival, and function, through binding to its CSF-R (receptor), which is expressed in most tumoral cells [33,35,36]. The CSF1 rearrangement is present only in 2–16% of tumor cells. These neoplastic TGCT cells overexpress CSF1 and, interestingly, also express CSF1R, resulting in their growth through an autocrine loop and in the recruitment and accumulation of nonneoplastic chronic inflammatory monocyte-like cells through a paracrine landscape effect [27,33,35,37].

Other genes, such as MMPS-1 and 9 and SSP1, are overexpressed, resulting in further inflammation and matrix degradation. The presence of pro-inflammatory cytokines (TNF, IL-1, IL-6) stimulate synoviocyte proliferation and MMPS leads to cartilage damage [30,38,39]. The synovial cells behave as osteoclast-like cells and produce high levels of RANKL, which contributes to osteoclast differentiation [39,40]. Trisomies 5 and/or 7 are also found in some cells, suggesting clonality and a neoplastic origin [41,42,43,44].

M-TGCT as a malignant neoplasm presents numerous (>20 mitoses per high-power field) and atypical mitoses, extensive necrosis, enlargement of the nuclei, a very large size of some cells ranging from 25 to 40 μm in diameter, an abundance of eosinophilic nuclei, and myxoid changes [1,44].

## 6. Principles of Treatment

The gold standard of treatment modality in TGCT remains surgical excision of the tumor but the high rate of recurrence and refractory disease, especially in D-TGCT, indicates the need for possible further treatment options [45].

### 6.1. Surgical Procedure

In N-TGCT, en block resection can be achieved with a low recurrence rate, particularly in extra-articular cases (like in the tendon sheaths of the hand or foot). In the case of intra-articular N-TGCT, specifically in the knee, a comprehensive study conducted by Mastboom et al. revealed a significant difference in recurrence rates between open and arthroscopic surgery (9% vs. 18%) [46].

The same study noted a higher rate of local relapse-free survival (LRFS) after open versus arthroscopic surgery in patients with N-TGCT in large joints, 87% versus 80%, respectively, but the statistical significance was lost in multivariate analysis [46]. In the anterior compartment, a mini open incision is preferable, while in the posterior compartment, open resection is recommended. Arthroscopy is considered the most optimal choice for treating issues in the shoulder or elbow [5].

For D-TGCT in the knee, in the anterior compartment, synovectomy might be achieved arthroscopically, while open synovectomy is preferred for the posterior compartment [47,48]. A systematic review revealed a lower recurrence rate after open synovectomy (14%) compared to arthroscopic synovectomy (40%) for D-TGCT in the knee [45]. Mastboom et al., in an international, retrospective study of patients with D-TGCT, reported a 5-year recurrence free-survival rate of 66% for open surgery and 54% for arthroscopic synovectomy [49]. Overall, surgery in recurrent-refractory disease has a remarkably higher risk of further local relapse rate and surgical treatment options in patients with D-TGCT are not a definite modality for every patient due to the high risk of local relapse and a relatively high risk of postoperative complications [49].

**Figure 3:**
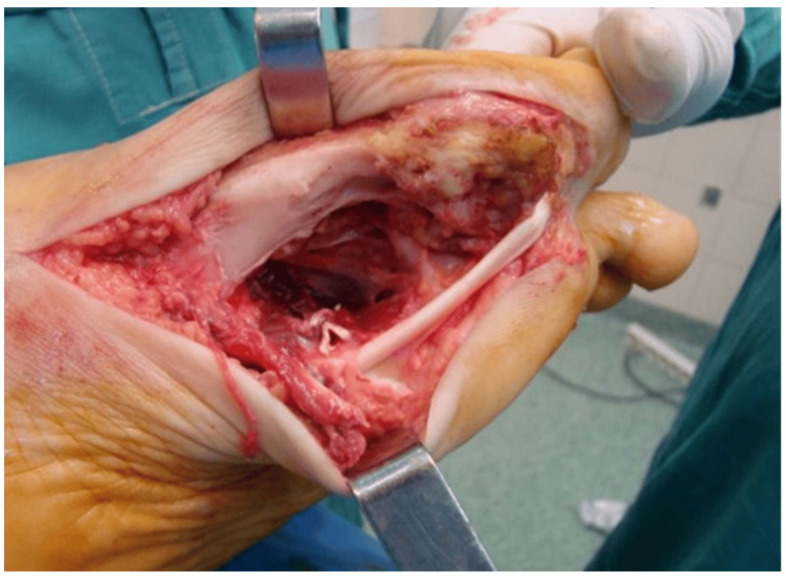
Intraoperative view of a foot following en block resection of a GCT TS.

**Figure 4:**
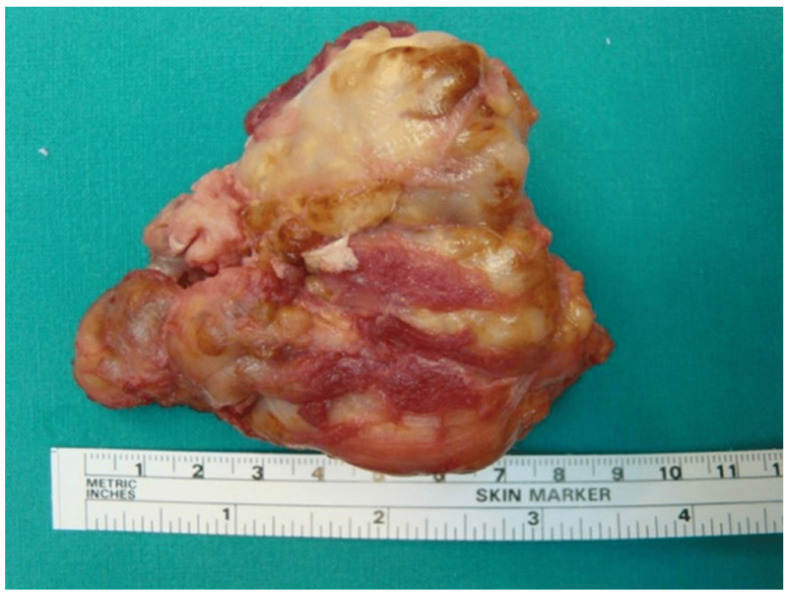
The resected specimen of the GCT tendon sheath.

Additionally, total arthroplasty may be considered in recurrent-refractory cases leading to degenerative secondary arthritis, as an option, not though as a first-line therapy. However, joint replacement has a low rate of local relapse, with better outcomes in the hip than when the knee is the affected joint [50,51] (Figure 3 and Figure 4).

### 6.2. Radiotherapy

Two forms of radiotherapy are available: external beam radiation (EBR) and radiosynoviorthesis (RSO).

EBR is indicated in inoperable disease and as adjuvant therapy to surgery in extra-articular disease, in relapsed D-TGCT, and in residual disease and the recurrence rate is between 6% and 13% [45,52,53,54,55,56]. EBR is also used as neoadjuvant therapy and is not advisable for hand and foot lesions [45]. The total doses that are recommended are 30–36 Gy and are delivered within 3–4 months of surgery [53,56]. Reported complications in association with EBR are poor wound healing, skin reactions, joint stiffness, femoral fractures, impotence, and sarcomatous transformation [31,56]. Therefore, EBR is not recommended for use routinely.

D-TGCT, RSO (radiosynoviorthesis), which is also called isotopic synoviorthesis and radiosynovectomy, consists of the intra-articular injection of 90-yttrium-labeled colloid and, as for EBR, is proposed just after surgery. The largest case series on RSO is a single-center report by Ottaviani et al. including 73 patients with TGCT who were treated with open synovectomy and additional RSO. After a mean follow-up of 4.6 years, the relapse rate was 30% for knee involvement and 9% for other joint involvement [15]. Severe complications have been described as radionecrosis and intra-articular infections [57]. Overall, RSO is not a therapeutic option for an insufficient surgical approach but it should be used as an adjuvant therapy after total synovectomy without residual disease and it may be effective in cases with large and relapsed D-TGCT [58].

This modality remains controversial regarding which in cases it could be used and could be effective and a main issue for experts is the complications of the radiotherapy, like fibrosis and joint stiffness and a long-term risk of malignant transformation.

### 6.3. Systemic Treatment

The search for understanding of the tumor biology and pathogenesis of TGCT led to the identification of a chromosomal translocation t(1;2) involving the ligand CSF1, which is overexpressed and attracts non-neoplastic cells expressing M-CSFR (mostly macrophages) through a paracrine landscape effect.

It is hypothesized that CSF inhibitors may disrupt the autocrine and paracrine loops, which are believed to be a major cause of TGCT growth [33,34,35].

In relapsed-refractory disease, especially in D-TGCT, new targeted systemic therapies are being used. A very important targeted treatment is the blockade of the CSF1/CSF1R signaling axis, achieved by blocking the receptor itself, the tyrosine kinase activity of CSF-1R using small inhibitory molecules (TKI) or monoclonal antibodies targeting CSF1-R. Another course of action to block this signaling axis is achieved by blocking the ligand CSF1 using antibodies [34] (Table 1 summarizes the treatment options).

### 6.4. Tyrosine Kinase Inhibitors

#### 6.4.1. Pexidartinib

Pexidartinib (Turalio) is an oral TKI selective CSF1-R inhibitor that targets CSF1R, c-cit receptor tyrosine kinase (KIT proto-ongogene), and FLT3 (fms-like tyrosine kinase 3 internal tandem duplication) [59] and was approved by the FDA in 2019 (but not by the European Medicines Agency) for adult patients with symptomatic TGCT associated with severe morbidity or functional limitation that was not amenable to improvement with surgery [60,61].

The ENLIVEN trial, a double-blinded, placebo-controlled Phase 3 study of pexidartinib, showed that patients receiving PLX3397 had a response rate of 39% at week 25, compared with 0% in the control group [60]. The overall response rates were even greater, at 56% for the RECIST (Response Evaluation Criteria in Solid Tumors), and 64% for the TVS (tumor volume score) (median follow-up, 22 months). Furthermore, Gelderblom et al. [62] reported overall response rates among pexidartinib-treated patients of 60% for the RECIST and 65% for the TVS.

This was a follow-up study that was extended 26 months after the ENLIVEN data cutoff [62].

Moreover, the study group showed amelioration in function with a notable increase in range of motion (ROM) from baseline when compared to the controls. Specifically, between baseline and week 25, the patients treated with pexidartinib had a mean improvement of 4.1 points in Patient-Reported Outcomes Measurements Information System-Physical Function (PROMIS-PF) scores (compared to the placebo patients, who had a mean decline of 0.9 points) that was maintained after 50 weeks of pexidartinib treatment [63].

However, pexidartinib has a generally manageable safety profile, except for hepatotoxicity. Specifically, pexidartinib has been associated with two types of hepatic adverse reactions: reversible ALT or AST growing and idiopathic mixed or cholestatic hepatotoxicity [64]. In the ENLIVEN study, 39% of patients in the pexidartinib cohort had AST elevations (10% were grade ≥ 3) and 28% experienced ALT elevations (10% were grade ≥ 3) and compared with the placebo group, the pexidartinib cohort had a higher rate of grade 3 or 4 adverse events (44% vs. 12%), including ALT, AST, and ALP elevations and hypertension [60]. Patients taking pexidartinib must have careful monitoring of liver function, particularly in the first 2 months of treatment. In patients with renal impairment, dosage adjustment is suggested.

Pexidartinib is a therapeutic option in patients with advanced disease for whom surgical management is not achievable or may lead to excessive morbidity. Further to that, pexidartinib might be an option in cases of refractory-relapsed disease. Following an initial course of 3–6 months of the therapy, the patient should be re-evaluated in case of refractory disease or as surgical candidates in case of initially unresectable disease.

Pexidartinib is not indicated in patients suffering from liver failure or injury, and who are on medications and have comorbidities that may impair liver function.

#### 6.4.2. Imatinib

Imatinib (GLEEVEC) inhibits multiple tyrosine kinases, against Alb, Bcr-Abl, c-KIT, PDGFRA, and CSF1R [65].

Blay et al. reported a complete response in a 34-year-old woman with recurrent TGCT after surgical excision who was treated with imatinib [66]. In a retrospective cohort of 29 patients with advanced or metastatic D-TGCT, Cassier et al. reported that 19% of patients had an overall objective response (1 patient had complete response and 4 had a partial response) and 74% had stable disease. In the same study, 73% of the patients achieved tumor functional and symptomatic improvement [67]. Interestingly, Stacchiotti et al. observed a response to imatinib in two patients with TGCT disease resistant to nilotinib [68].

Verspoor et al., in a retrospective study with a long follow-up of patients with locally advanced, recurrent diffuse TGCT treated with imatinib, confirmed an overall response rate (ORR) of 31% with a disease control rate of 96% at a mean follow-up of 52 months [69]. Specifically, 4% of patients showed a complete response (CR), 27% showed a partial response (PR), and 65% had stable disease (SD). Adverse events included fatigue, edema/fluid retention, and nausea. Serious adverse events, grade 3–4 toxicities, were noticed in 11% of the patients treated with imatinib and 12% of patients discontinued treatment due to toxicities [69].

In another study including 25 patients with locally advanced or recurrent D-TGCT treated with imatinib, Mastboom et al. [70] assessed the effect of imatinib pre- and post-therapy by comparing MRI scans and PET-CT. MRI assessment of the involved joints showed a significant mean difference of 12% in the TVS (tumor volume score) between the pre- and post-imatinib scores and PET-CT showed a significantly decreased mean difference of 5.3% SUV-max between the pre- and post-treatment values in patients treated with imatinib. Overall, this study confirmed the moderate radiological response of imatinib in D-TGCT and the value of PET-CT as a diagnostic tool [70].

Imatinib still remains an option in recurrent disease after surgery based on estimates of its favorable safety profile and the durable effect after discontinuation [69].

#### 6.4.3. Nilotinib

Nilotinib (TASIGNA) inhibits several tyrosine kinases including PDGFR-alpha, c-KIT, ABL, and CSF1R [71]. A multicenter, open label, single-arm, phase 2 trial investigated the efficacy and safety of nilotinib in patients with locally advanced, relapsing inoperable D-TGCT [72]. Gelderblom et al. reported that the assessed proportion of patients who were progression-free at 12 and 24 weeks was 92.6% and 90%, respectively. No patients achieved an objective response or complete response at week 12 and after one year of treatment and follow-up 90% showed stable disease {SD} and 6% achieved a partial response (PR). Common adverse events were headache, dizziness, hepatic disorders, and fatigue [72].

In a long-term follow-up of nilotinib in patients with D-TGCT, Spierenburg et al. found that 6.3% of the patients achieved a partial response (PR) as the best overall response and 93.8% achieved stabilization of the disease. Also, 52% of the patients had progression and the five-year PFS (progression-free survival) rate was 53% [73].

Nilotinib is under study in a randomized clinical trial NCTO2029001 [5].

Nilotinib is an alternative strategy for patients with advanced non-amenable to surgical resection D-TGCT or in cases of relapse. However, its suitability for intermittent usage appears limited since only 6% of the patients attained a partial response and almost half of the patients showed progression and clinical worsening [73]. It has to be mentioned that other patients had ongoing disease control following a brief treatment period, indicating long-term efficacy [73].

#### 6.4.4. Vimseltinib

Vimseltinib (DCC-3014, Deciphera) is an oral switch-control tyrosine kinase inhibitor, designed to selectively and potently inhibit CSF1R, binding to CSF1 receptors with a specificity of more than 100-fold versus all kinases tested and is > 500-fold selective versus other similar kinases such as FLT3, PDGFRA, PDGFRB, and KIT [74,75]. Smith et al. showed that vimseltinib, in preclinical studies, depleted TAMs (tumor-associated macrophages), CD16+ monocytes, and other CSF1R-dependent cells and resulted in the inhibition of tumor growth and bone degradation [74].

Gelderblom et al. investigated the safety and preliminary efficacy of vimseltinib received by patients with TGCT not amenable to surgery. The majority of adverse events were grade 2 and lower and the observed transaminase, pancreatic, and CPK enzyme elevations were mostly low grade. In the phase 1 cohort, a high ORR of 50% was observed and in the phase II cohort, the patients achieved an ORR of 42% (all partial responses) [76].

Tap William et al. reported a statistically important and clinically remarkable improvement in the 25-week overall response rate vs. placebo in patients with TGCT not amenable to surgery in the MOTION study, a randomized, phase III study of vimseltinib [77,78]. The phase III MOTION trial showed that patients who were treated with vimseltinib achieved a 25-week ORR of 40% vs. 0% in the placebo group. The study met also all key secondary endpoints, including a 25-week ORR by tumor volume score (TVS) of 67% with vimseltinib vs. 0% with placebo and an improvement of 18.4% in ROM at week 25 with vimseltinib vs. 3.8% with placebo [78].

Vimseltinib has been shown in data reviews to potentially become a new treatment option for patients with TGCT and recurrent disease due to its significant efficacy and its safety profile [77,78].

#### 6.4.5. Emactuzumab

Emactuzumab (RG-7155) is a recombinant, humanized monoclonal antibody against CSF1R expressed on macrophages and represents another way to block the CSF1/CSF1R axis [79].

In a phase 1 trial, Emactuzumab showed clinical activity in locally advanced or relapsed D-TGCT and a safety profile. In total, 86% of patients treated with emactuzumab achieved an objective response and 7% achieved a complete response. Most of the adverse events were grade 1 and 2 and the most frequently reported were pruritus (56%), asthenia (56%), facial edema (64%), and peripheral edema (36%) [80].

In an open-label phase 1 study of 63 patients with D-TGCT who received emactuzumab, Cassier et al. reported that the overall objective response rate (ORR) was 71%. Also, they identified that the responses were durable and an ORR of 70% and 64% was determined one or two years after enrolment into the study. Clinical activity was accompanied by an amelioration in EuroQol-5D-3L and particularly the joint disorder-specific WOMAC score [81].

In a third study, Smart et al. showed that the optimal biological dose (OBD) of emactuzumab for q2w dosing was >=900 mg, approximately three-fold lower than the highest dose tested clinically [82]. In the extension phase of a phase I study, an OBD of 1000 mg/i.v q2w was recommended [80]. Finally, this study identified that dosing flexibility is possible by dosing with emactuzumab once q3wks [82].

Emactuzumab appears promising and is generally well tolerated, with asthenia, facial/periorbital/eyelid edema, and pruritus being the most frequent adverse events, and thereby exhibits a favorable comparison to other CSF1R-targeting agents [80,81,82]. Emactuzumab could be used as neoadjuvant or adjuvant treatment (pre- or post-surgery in relapsed TGCT) and demonstrates a significant clinical response after a short duration of treatment of four or five cycles [81]. However, further studies on the optimal duration and the long-term effects of the emactuzumab are needed.

#### 6.4.6. Cabiralizumab

Cabiralizumab (FPA-008, Cabira), is an intravenous monoclonal antibody that inhibits the interaction of the CSF1 and IL-34 ligands with their receptor CSF1R [83]. This leads to the reduced stimulation and survival of TAMs (tumor-activated macrophages) and monocytes.

In an I/II study (NCT02471716), Sankhala et al. evaluated the safety and efficacy of cabiralizumab administered i.v. every 2 weeks for 6 months in patients with D-TGCT. In phase 1, 38 patients received 1 mg/kg (*n* = 3), 2 mg/kg (*n* = 3), and 4 mg/kg (*n* = 32), following a 3 + 3 dose escalation design. Five patients who received 4 mg/kg showed a PR and the patients who received a 1 and 2 mg/kg dose did not show any response. No dose-limiting toxicity was identified and the most common adverse events were creatine kinase elevation, rash, periorbital edema, and hypertension. In phase 2, the ORR (objective response rate) was 25% for 4 mg/kg cabiralizumab for up to 12 doses and 33.3% for 4 mg/kg cabiralizumab on days 1 and 15 of cycle 1 and then every 4 weeks up to 12 months [83].

Cabiralizumab has been investigated in non-operable TGCT disease or tumors for which resection would cause severe morbidity. However, this agent would be administrated in patients with relapsed TGCT, as it has exhibited clinical improvement and a radiographic response [83] but further investigation will be necessary for long-term efficacy.

#### 6.4.7. Lacnotuzumab

Lacnotuzumab (MCS-110) is another recombinant, humanized, intravenous monoclonal antibody against CSF1 and has been tested in a double-blind, randomized phase Ib/II study. The preliminary results of this study were presented during a congress [84]. In the extension of the study, 7 patients received multiple monthly doses of 3 mg/kg, 7 patients received multiple doses of 5 mg/kg, and 15 patients received a high dose of 10 mg/kg. After a single dose, the tumor size decreased by 7.4% in the low-dose group (3 mg/kg), by 25% in the medium-dose group (5 mg/kg), and by 33% in the high-dose group (10 mg/kg). After multiple doses, the tumor size shrank by 30%, 56%, and 55% in each group, respectively. Lacnotuzumab was well tolerated and was safe overall, while adverse events were mild and uncommon. Study results are pending (NCT01643850) [85,86].

Lacnotuzumab could be another therapeutic approach for refractory and relapsed TGCT, as it has the advantage of a favorable safety profile.

#### 6.4.8. Pimicotinib

Pimicotinib (ABSK-021) is an oral daily (50 mg) medication and selective small molecule antagonist of CSF1-R with minimum inhibition of c-Kit and PDGFR [87].

A phase 3 study, known as MAEUVER, has investigated the effectiveness and safety of this drug. After 6 months of treatment with pimicotinib, TGCT patients showed improvement in pain, stiffness, and range of motion and 77.4% of patients had more than a 30% shrinkage of the tumor. The most common adverse effects were CPK and transaminase elevations, which quickly recovered after drug interruption. Serious liver injuries were not reported [87].

A phase 3 trial of this medication is ongoing [87].

Pimicotinib will be an option in the future as a neoadjuvant therapy or in refractory TGCT disease since it has demonstrated significant antitumor activity and favorable safety.

#### 6.4.9. Sotuletinib

Sotuletinib (BLZ945) is a highly effective, selective and brain-penetrating inhibitor of CSF-1R (c-Fms).

Thongchot et al. established four novel cell lines isolated from the tissue of the primary tumor of patients with TGCT. All the TGCT cells expressed a high level of CSF1R and the treatment with sotuletinib showed a significant inhibition of TGCT cell growth and induced cell apoptosis correlated with the CSF1R level. So, further investigation needs to be achieved [88].

#### 6.4.10. Anti-TNF Blockade

##### Infliximab

Both TGCT and rheumatoid arthritis are described by the presence of activated macrophages and the expression of pro-inflammatory cytokines such as TNF-a, which promotes the differentiation and activation of osteoclasts and stimulates synovial cells to secrete MMPs. There is a common autocrine mechanism in osteoclast differentiation in both diseases [38,89]. Also, a synergic paracrine loop mediated by TNF-a and CSF1, which is overexpressed in TGCT disease, has been involved in both inflammatory and neoplastic conditions [90,91]. So, using TNF-blockers may have a potential role in therapy for TGCT.

So far, treatment with a TNF-a blockade in TGCT patients is presented as a case report in 2005. Kroot et al. [92] reported on a man aged 22 years with refractory TGCT in the right knee, who underwent open surgical synovectomy and who, following intra-articular injections of yttrium-90, received an anti-TNF-a monoclonal antibody i.v. (infliximab) at a starting dose of 5 mg/kg and was then given it at 2, 6, 14, and 20 weeks and bimonthly for up to 54 weeks. The patient responded well to this treatment without any side effects and showed significant clinical and histological improvement with a remarkable reduction in macrophages and pro-inflammatory cytokines [92].

Praino et al. [93] reported good results in three patients with recurrent TGCT in the knee after surgical synovectomy, who were treated with an intra-articular injection of 100 mg of infliximab within 12 months. Two of them underwent subsequent synovectomy and achieved complete remission at 12 months and the third one refused surgery and remained on local infliximab therapy and in remission. No adverse events or local reactions were detected [93].

##### Adalimumab

Adalimumab is another monoclonal antibody against TNF-a. Kobak et al. [94] reported a significant clinical and radiological improvement in a patient with TGCT in the knee, who underwent an intra-articular injection of Adalimumab. The patient did not consent to surgical intervention and in total was administered four intra-articular doses of 40 mg Adalimumab. At the six-month follow-up visit, the patient was still in remission and in complete response [94].

##### Etanercept

Etanercept is a human tumor necrosis factor receptor p75 Fc fusion protein using recombinant DNA technology and tested in a Chinese hamster ovary mammalian expression system. Fiocco et al. [95] reported the case of two patients with recurrent D-TGCT in the knee, who were treated with intra-articular injections of etanercept and presented significant improvement in knee disease activity and maintained functional recuperation. They received 12.5 mg weekly IA-ETN injection for 4 weeks, followed by extended arthroscopic synovectomy and 25 mg IA-ETN injection for 4 weeks.

Extensive expertise exists on the application of anti-TNF-alpha inhibitors in the management of many rheumatic diseases, which is advantageous given their safety profile. These agents may serve as an alternate option for patients with relapsed-refractory TGCT following surgical resection or as neoadjuvant treatment prior to surgery.

##### Bevacizumab

Bevacizumab (Avastin) is a humanized monoclonal anti-vascular [NS1] endothelial growth factor (VEGF) antibody and thus inhibits angiogenesis, which is crucial in tumor development and is induced by CSF1, which is overexpressed in TGCT disease [96,97].

It has been used in a patient with recurrent D-TGCT in the knee after debulking arthroscopic synovectomy. Intra-articular injections of 100 mg Bevacizumab were administrated to the patient repeated monthly for 12 months; the patient achieved significant clinical and imaging responses, as described by Nissen et al. [98]. At follow-up 2 months after the final injection, the patient presented a complete response with no pain and no limitation of the knee range of motion. No adverse events were observed [98].

Antiangiogenic agents such as Bevacizumab may serve as a potential therapeutic target by regulating the enhanced vascularity in tumors, for the treatment of resistant D-TGCT, or as neoadjuvant therapy for inoperable TGCT. The drawback of this method is the absence of additional data regarding long-term efficacy in TGCT.

##### AMB-05X

AMB-05X is a human immunoglobulin IgG2 monoclonal antibody against CSFR1. In a phase II trial (NCT04731675), 150 mg AMB-05X is administered as a joint injection to the knee every two weeks for 12 weeks [99].

This is an ongoing investigation for this agent.

##### Zaltoprofen

Ζaltoprofen is a nonsteroidal anti-inflammatory drug that inhibits the augmentation of TGCT stromal cells via activation of PPARγ. Zaltoprofen also has been reported to cause apoptosis in rheumatoid synovial cells via PPARγ [100,101]. PPARγ is a ligand-activated transcription factor and is included in the nuclear hormone receptor superfamily. It is a key transcriptional factor that promotes adipocyte differentiation and has antitumor activity by inhibiting tumor proliferation and through apoptosis [100,101].

Takeuchi et al. [102], in a randomized placebo-controlled double-blind phase II study of zaltoprofen for patients with D-TGCT and unresectable localized-TGCT, reported a significant improvement in physical function following zaltoprofen treatment (480 mg/day p.o) at 48 weeks but no significant differences in the PFR (progression-free rate) between the two groups (placebo and zaltoprofen). At 48 weeks, eight patients presented stable disease and one showed progressive disease at 72 weeks [102]. Zaltoprofen was well tolerated. This is a novel therapeutic option for TGCT but further investigation of the long-term administration of zaltoprofen should be considered.

## 7. Conclusions

TGCT is a benign tumor with inflammatory features and a destructive and aggressive course that can lead to significant morbidity and functional impairment with a high impact on quality of life.

The tumor is driven by a chromosomal translocation, t(1;2)(CSF-1;COL6A3), in the majority of cases, which is present in 2–16% of tumor cells, leading to the overexpression of CSF1 and recruitment of CSF1R macrophages, giant cells, and osteoclasts.

Surgical resection remains the gold standard current treatment and the optimal surgical approach depends on the location and extent of the tumor. However, D-TGCT has a significantly high rate of recurrence.

In refractory disease of TGCT, radiotherapy could be a therapeutic option but is rarely used due to its low efficacy and high risk profile for complications and malignant transformation.

Novel systemic therapies have been recently used in relapsed D-TGCT. The development of new targeting drugs that block the CSF1/CSF1R signaling axis is a significant treatment modality. An interdisciplinary approach with surgical–orthopedic oncologists, medical and radiation oncologists, and physical therapists, cooperating in the management of recurrent-refractory D-TGCT, will be vital. Several questions remain about the optimal therapeutic approach in refractory disease or the optimal treatment duration. Understanding more about the biology and the molecular mechanisms of this oncogene-driven tumor will help to develop new targeting systemic therapies and the results of current clinical trials will help us to understand the role of these medications.

## Figures and Tables

**Table 1:** Indications, benefits and drawbacks of current available treatment options.

Treatment Option	Indications	Benefits	Drawbacks
Pexidartinib	Approved by FDA for TGCT	Significant overall response rate 56% by RECIST at wk 25	Hepatotoxicity
Imatinib	Recurrent TGCT	Safe profile	45 = CR, 27% = PR
Nilotinib	Neoadjuvant, Relapsed TGCT	Safe profile, Ongoing effect after discontinuation	6.3% = PR, 52% had progression
Vimseltinib	Recurrent TGCT	Safe profile, ORR = 40% at 25 wk	
Emactuzumab	Neoadjuvant, Adjuvant Recurrent TGCT	ORR = 71%, Durable response, Safe profile	7% = CR
Cabiralizumab	Recurrent TGCT Neoadjuvant Adjuvant	Safe profile, 33.3% = ORR (12 m)	
Lacnotuzumab	Recurrent TGCT	Safe profile, Tumor size shrunk by 55% (10 mg/kg)	
Pimicotinib	Recurrent TGCT	77.4% had more than 30% tumor shrinkage	
Sotuletinib			
ANTI-TNF Inhibitors	Recurrent TGCT	Safe profile	
Bevacizumab	Recurrent TGCT Neoadjuvant	Safe profile	
AMB-05X		Safe profile	
Zaltoprofen	Recurrent TGCT	Safe profile	
